# Advances in Sustainable Polymeric Materials

**DOI:** 10.3390/polym14224972

**Published:** 2022-11-17

**Authors:** Cristina Cazan

**Affiliations:** Renewable Energy Systems and Recycling Research Center, Transilvania University of Brasov, 500036 Brasov, Romania; c.vladuta@unitbv.ro

Sustainable polymeric materials are materials of great technological importance and are specially created to meet unique demands regarding: mechanical resistance and rigidity; corrosion resistance; resistance to the action of chemical agents; low weight; dimensional stability; resistance to variable stress, shock and wear; insulating properties; and aesthetics. These characteristics have led to the use of composite materials on an increasingly large scale and stimulated research to discover new types of composite materials with improved properties. Major concerns and realisations of high-performance composite materials exist in all developed countries, due to the desire for continued technological development and the use of qualitatively superior materials achieved through efficient and non-polluting processes and technologies.

This Special Issue includes twenty-two articles highlighting advances in virgin and waste polymeric materials, mainly advancements in the research and development of synthesis, characterisation, processing, morphology, structure, properties and applications for nanocomposites and hybrid polymer composites, blends and polymeric materials for sustainability.

Below is a brief summary of the papers included in this issue, considering the types of advances in sustainable polymeric materials used with applicability in different fields of activity.

In the actual contest of the environment’s care and pollution reduction, biodegradable plastics obtained from renewable sources represent a great challenge for the research domain. Xuan Tan et al. [1] highlight the most significant achievements in this research direction, particularly on the role of nanofillers and plasticisers utilised in bioplastic fabrication, focusing on starch-based bioplastics. Xuan Tan et al. [2] optimised a rapid ultrasound-assisted starch extraction from sago pith waste (PSW) to fabricate a sustainable bioplastic film. This process was proven to be more efficient than conventional extraction economically and potentially sustainable for the production of bioplastic film. The ultrasound-extracted sago starch was used to prepare a bioplastic film via the solution-casting method. The article [3] traces a necessary and current state of the art on bioplastics, coherently addressing concepts, classifications, production chain, biodegradability and compostability standards, LCA of bioplastics, and finally, a summary of opportunities and possible challenges. Ungprasoot et al. [4] obtained bioplastic from waste biomass using water hyacinths, bagasse and rice straw via extraction techniques. First, it was synthesised carboxymethylcellulose (CMC) and then used for biopolymer production, using tapioca starch solution and glycerol as a binder and an additive. Biopolymers obtained can be an alternative material to develop into food and drink packaging, being an environmentally friendly product. At the same time, these can be naturally decomposed in a short time, leading to a reduction in pollution from petroleum-based plastics.

Magazzini et al. [5] presented a process of obtaining physical mixtures of biodegradable polymers poly (glycolic acid) (PGA) with poly(L-lactide) (PLLA) and polycaprolactone (PCL) using the melt blending technique. Their morphology, wettability, thermal properties, and degradation behaviour were analysed using SEM, contact angle, DSC, and TGA. Their results show that PGA affected polymer matrices’ thermal and degradation behaviour and accelerated their degradation, a trait desirable for creating biodegradable and compostable plastics while not increasing the cost substantially.

Naser et al. [6] reviewed modification techniques on the mechanical properties of PLA and PHAs. The main aim of this review article is to widen the applicability of both biopolymers so that they can eventually replace petroleum-based plastics in new potential applications and therefore reduce the amount of waste and pollution.

Recent research studies demonstrated the tremendous potential biodegradability of both thiophene-based homopolymers and copolymeric systems, opening a new method for the industrial recycling of furan and thiophene-based materials. Thus, Guidotti et al. [7] synthesised polycondensation materials suitable for the realisation of rigid and flexible films for packaging applications.

The CarbaCell process is a promising alternative to produce recycled cellulose filaments, membranes, aerogels, and clothing fabrics, using cellulose carbamate as an active intermediate for fibre spinning. The industrialisation of this process is significant for the environment and economic development. Kang et al. [8] detailed the dissolution process of CarbaCell, and the effects of different NaOH and ZnO contents on the dissolution of CC were compared.

The most recent method of valorising recycled polymeric materials is their use of obtaining materials used in the construction field being a low-cost and somehow environmental beneficial solution. Nizamuddin et al. [9] presented the benefits of polymer-modified bitumen and some limitations and challenges that need to be considered when using virgin and recycled plastic to enhance bitumen performance. Different polymers such as HDPE, LDPE, LLDPE, MDPE, PP, PS, PET, EMA, and EVA have been successfully employed for bitumen modification. Different characteristics of plastics-modified bitumen, including chemical, thermal, rheological, structural, and mechanical properties, were investigated.

Cosnita et al. [10] presented the development of new value-added composite materials based on only waste: recycling tire rubber, polyethene terephthalate (PET), high-density polyethene (HDPE), wood sawdust, and fly ash. The influence of fly ash on the mechanical properties and water stability of the new “all-waste composites” was assessed, considering their applications as outdoor products in the construction field.

Sukhawipat et al. [11] developed a novel rigid sound-absorbing material made from used palm oil-based polyurethane foam (PUF) and water hyacinth fibre (WHF) composite. This material might be a promising candidate for the sound-absorbing material industry due to its high efficiency with an NCO index of 100 and 1%wt of WHF, mechanical characteristics, flammability and sound absorption.

Komartin et al. [12] presented a relevant study to obtain new sustainable composites based on epoxidised linseed oil and kraft lignin as anticorrosive coatings. The structure, thermal stability, dynamic mechanical behaviour and corrosion protection of carbon steel were investigated. The obtained bio-based epoxy-lignin coatings can mitigate the corrosion processes for carbon steel.

Recent advances in obtaining polymeric materials by improving the interfaces (of inorganic/organic nature) using different fillers and coupling agents have shown strong potential to generate materials. Nanocomposites with polymer matrix were discussed in many studies. Still, Cazan et al. [13] offer excellent opportunities to explore new functionalities of TiO2 as a reinforcement agent in polymeric nanocomposites beyond those of conventional materials. This review aims to provide specific guidelines on the correlations between the structures and properties of TiO_2_ nanocomposites, established and explained based on interfaces realised between the polymer matrix and inorganic filler.

Enesca et al. [14] realised a critical presentation of the photocatalytic activity of TiO_2_, ZnO, WO_3_, Fe_2_O_3_, and Bi_2_MoO_6_ from polymer composites against different organic compounds (dyes, active pharmaceutical molecules, phenol, etc.). The effect of other polymeric composites and photocatalytic parameters on the overall photocatalytic efficiency is described. Representative studies were included and correlated with outlining the significance of polymeric composite composition and testing parameters on the photocatalytic removal of pollutants.

Lamparelli et al. [15] present a relevant study to demonstrate the processability and characterisation of sustainable elastomers by anionic copolymerisation of renewable terpenes in a wide range of compositions with interesting thermal profiles and different polymeric architectures by simply modulating the alimentation feed and the [monomers]/[initiator systems] ratio. Thus, homo- and co-polymerizations of myrcene with styrene and isoprene and terpolymerization of all monomers have been reached using sodium hydride in combination with triisobutylaluminum as the anionic initiating system at 100 °C in toluene.

Wang et al. [16] conducted a systematic study to understand the interactions between vanillyl alcohol-based epoxy better and cross-linkers across-linkerserties of the cured epoxy systems. The authors emphasise the interactions between molecular structure and functionality of the cross-linker and the vanillyl alcohol epoxy resin during the curing process aspects beneficial for optimising bioepoxy formulations targeting industrial applications.

The acquisition of rare earth elements is significant from the point of view of environmental protection and the current state of natural resources. In this regard, Allam et al. [17] reported synthesising and characterising a new polymeric composite based on polyvinyl chloride (PVC). RE ions’ desorption/uptake capacity from loaded cetylpyridinium bromide/polyvinylchloride (CPB/PVC) sorbent was investigated. High-extract rare earth elements (REEs) uptake values were achieved, and the optimal sorption conditions were thoroughly investigated.

Möhl et al. [18] provided a comparative study of using lignin-based thermoplastic on commercial flex yarn to improve the tensile properties compared to the flex yarn itself.

Onffroy et al. [19] presented an effective chemical foaming process of polylactic acid (PLA) via the solid-state processing methods of solid-state shear pulverisation (SSSP) and cryogenic milling. The effects of the pre-foaming solid-state processing method and chemical foaming agent (CFA) concentration were investigated.

Polymer materials are also widely used in the medical field. Nowadays, the use of essential oils has increased. Essential oils (EOs) are complex polymeric mixtures of volatile compounds extracted from different parts of plants by other methods. Sousa et al. [20] presented different microencapsulation strategies for general and essential oils, some extraction methods for essential oils, and their applications. Khan et al. [21] highlight an essential issue for the safety and protection of human health, namely, timely and accurate glucose detection and monitoring. The authors developed sensitive and reliable electrochemical sensors for glucose determination to solve this problem using affordable and readily available materials. Thus, a non-enzymatic glucose electrochemical sensor based on an electrochemically synthesised PANI/Bimetallic oxide composite was reported. Polyaniline was synthesised by the electrochemical method, more precisely, the chronoamperometric method. Bayan et al. [22] synthesised a smart drug delivery system based on pH-sensitive polymeric formulations using a free radical bulk polymerisation method with different monomer and crosslinker concentrations. The optimisation of this smart system was investigated to achieve a colon-specific drug delivery, thereby improving the therapeutic efficacy and reducing the dosing frequency and potential side effects.

As the editor of this Special Issue, I recognise that the diversity and innovation of new sustainable polymeric materials rapidly developing in the multidisciplinary research field, sometimes using waste as raw materials, cannot be collected in a single volume. However, I am sure that this collection will contribute to the interest in the research in this area, providing our readers with a broad and updated overview of this topic.
